# A Case of Stage III c Ovarian Clear Cell Carcinoma: The Role for Predictive Biomarkers and Targeted Therapies

**DOI:** 10.3390/ijms14036067

**Published:** 2013-03-15

**Authors:** Munmun Rahman, Kentaro Nakayama, Tomoka Ishibashi, Masako Ishikawa, Mohammed Tanjimur Rahman, Hiroshi Katagiri, Atsuko Katagiri, Kouji Iida, Yoshihiro Kikuchi, Kohji Miyazaki

**Affiliations:** 1 Department of Obstetrics and Gynecology, Shimane University School of Medicine, Izumo, Shimane 6938501, Japan; E-Mails: munmun_dmc@yahoo.com (M.R.); tomo0314@med.shimane-u.ac.jp (T.I.); pcbashi4@yahoo.co.jp (M.I.); tanjim_dmc@yahoo.com (M.T.R.); jimipin999_lessismore@yahoo.co.jp (H.K.); atsuko_shimane@yahoo.com (A.K.); iida@med.shimane-u.ac.jp (K.I.);miyazaki@shimane-u.ac.jp (K.M.); 2 Department of Gynecology, Ohki Memorial Kikuchi Cancer Clinic for Women, Tokorozawa, Saitama 3591133, Japan; E-Mail: qwl04765@nifty.ne.jp

**Keywords:** ovarian clear cell carcinoma, biomarkers, PIK3CA, mTOR inhibitor

## Abstract

Ovarian cancer treatment presently does not reflect molecular differences in histologic subtype. Ovarian clear cell carcinoma (OCCC) exhibits several differences in terms of molecular pathogenesis and tumor behavior from the more common, chemosensitive, serous carcinomas, which makes OCCC a candidate for targeted therapies. A 53-year-old Japanese woman was diagnosed with stage IIIc ovarian clear cell adenocarcinoma with marked chemoresistance to conventional regimens. She demonstrated a partial response to a multikinase inhibitor. The tumor was resistant to PI3K/mTOR pathway inhibitors despite harboring a *PIK3CA* mutation. The present case suggests a role for targeted therapies in the treatment of OCCC and a need for the identification of biomarkers that will predict response to targeted therapies.

## 1. Introduction

Unlike the more common serous ovarian cancers, ovarian clear cell carcinoma (OCCC) is more frequently resistant to conventional platinum/taxane chemotherapy, which worsens its prognosis [1,2]. Therefore, a need exists for subtype specific therapies. Current clinical trials evaluating targeted therapies, however, are not histological type-specific. Here we describe a case of stage IIIc chemoresistant ovarian clear cell adenocarcinoma, in which a multikinase angiogenesis inhibitor achieved a partial response. This patient was a participant in a previously reported preclinical study [3,4].

## 2. Case History

**Figure 1:**
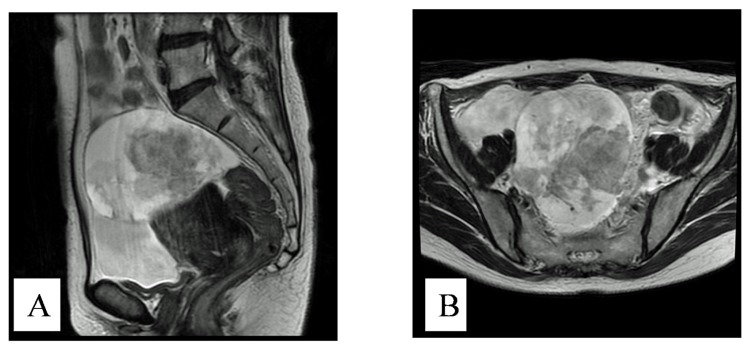
Enhanced magnetic resonance imaging (MRI) showing an irregular right adnexal mass, ascites. (**A**) Preoperative sagittal and T2-weighted images; (**B**) Preoperative axial and T2-weighted images.

**Figure 2:**
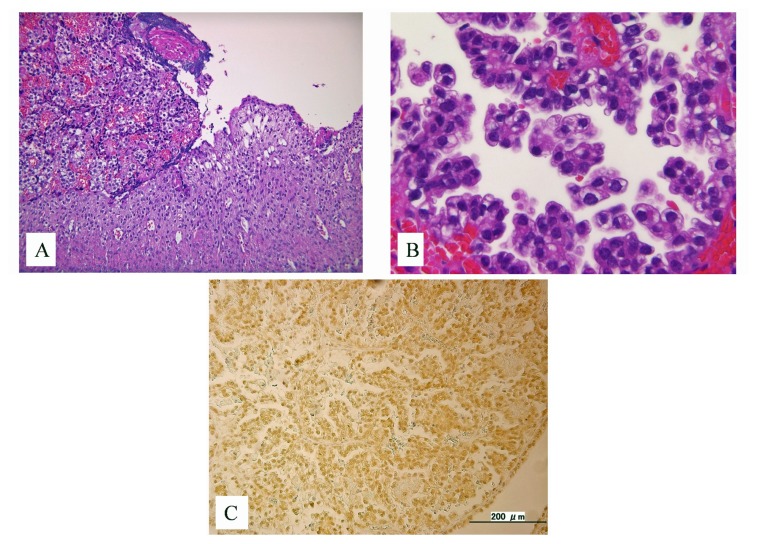
(**A**) Histopathological evidence of the transition from endometriosis to clear cell carcinoma; (**B**) The typical hobnail cells of clear cell adenocarcinoma; (**C**) High expression of hypoxia inducible factor 1 α (HIF1α) observed in the ovarian clear cell carcinoma cell nucleus.

A 53-year-old postmenopausal woman was referred from her primary care provider for suspected ovarian cancer. She had a prior history of endometriosis. The physical examination revealed massive ascites and palpable disease in the pouch of Douglas. The CA125 level was 604 ng/mL. A preoperative MRI showed a 9.8 × 9.0 × 84 mm, irregular, right adnexal mass and ascites (Figure 1). Subsequently, she underwent a primary debulking surgery which included a total abdominal hysterectomy, bilateral salpingoophorectomy, omentectomy, pelvic lymph node dissection, and low anterior colon resection. She was optimally cytoreducted in microscopic level. The histopathological findings revealed stage IIIc right ovarian carcinoma (clear cell type). There was also evidence of transition to clear cell carcinoma from within areas of endometriosis (Figure 2). There were metastases to the surface of the left ovary, mesenteric lymph nodes and omentum. The tumor carried both a *PIK3CA* and an *ARID1A* mutation [3,4].

She was scheduled for six cycles of adjuvant chemotherapy with irinotecan and cisplatin. Her disease free interval was three months. She recurred with a single diaphragmatic lesion measuring >4 cm adjacent to the left lobe of liver. She underwent a secondary debulking surgery and received three cycles of carboplatin and paclitaxel followed by liposomal doxorubicin for three cycles as well as liposomal doxorubicin combined with gemcitabine, all with progression as evidenced by a rising CA125, re-accumulation of ascites, and the development of new metastatic lesions (metastasis to a supradiaphragmatic lymph node, liver, and splenic hilum).

The patient was deemed a candidate for targeted/biologic therapy. Following a written consent process she was treated with the combination of bevacizumab, oxaliplatin, gemcitabine and sorafenib at Ohki Memorial Kikuchi Cancer Clinic for Women. As the targeted agents had not been approved by the Japanese Ministry of Health, Labour, and Welfare for the treatment of ovarian cancer, the patient bore the cost of her medications.

**Figure 3:**
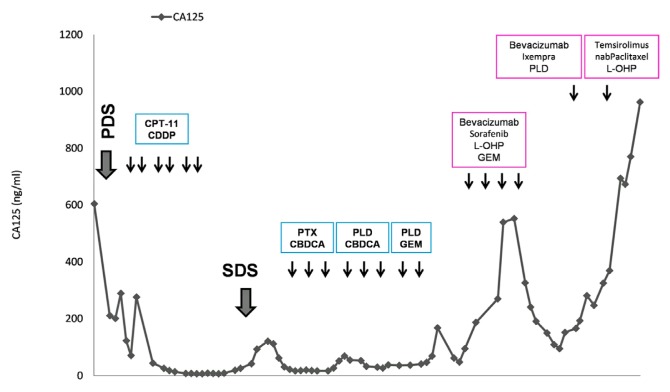
CA125 (Cancer Antigen 125) levels across the treatment course.

During the first cycle, the patient experienced an acute ischemic stroke, likely the result of cancer-associated venous thromboembolism (Trousseau syndrome), from the elaboration of excessive tissue factor [5]. She responded well to treatment and regained a sufficiently good functional status to resume chemotherapy. Following three additional cycles, she demonstrated a partial response in terms of a decrease in CA125 and a reduction in ascites (Figure 3). She was unable to receive her fifth cycle as she developed grade 3 acral erythema of the hands and feet, attributable to sorafenib. She was switched to the combination of bevacizumab, ixabepilone, and doxorubicin; however, both her tumor deposits and ascites increased. She was then started on temsirolimus, oxaliplatin and nab-paclitaxel with no response. She eventually died of her disease two years following her diagnosis.

## 3. Discussion

Ovarian clear cell carcinoma (OCCC), akin to a type I ovarian cancer [6], has a unique morphology characterized by glycogen containing clear cells and “hobnail” cells (Figure 2B). These tumors have recently been shown to arise from atypical endometriosis in about 49% of cases [7] (Figure 2A).

The genetic evaluation of the present case identified mutations of both *ARID1A* and *PIK3CA* (Table 1) as previously reported [3,4]. A somatic inactivating mutation of *ARID1A* (50% of cases) and an activating mutation of *PIK3CA* (33%–37% of cases) are the most common molecular genetic changes identified in OCCC [6]. In addition, single nucleotide polymorphism (SNP) array analysis has identified frequent amplification of the *ZNF217* (zinc finger protein 217) locus and deletion of the *CDKN2A/2B* locus in OCCC [6]. These changes distinguish OCCC from the more common, chemosensitive serous carcinomas, which more frequently harbor alterations in *P53*[6]. Despite the identification of these differences in molecular pathogenesis and tumor behavior, therapeutic approaches to ovarian cancer are, for the most part, non-targeted. Early disease is generally treated with a detailed staging laparotomy, whereas advanced disease is treated with optimal tumor debulking followed by adjuvant platinum/taxane chemotherapy. While the initial response to platinum/taxane chemotherapy in serous carcinomas approaches 80%, OCCC are in most cases chemoresistant [1,2]. There exists a significant need to develop therapies targeting the unique biology of OCCC.

**Figure 4:**
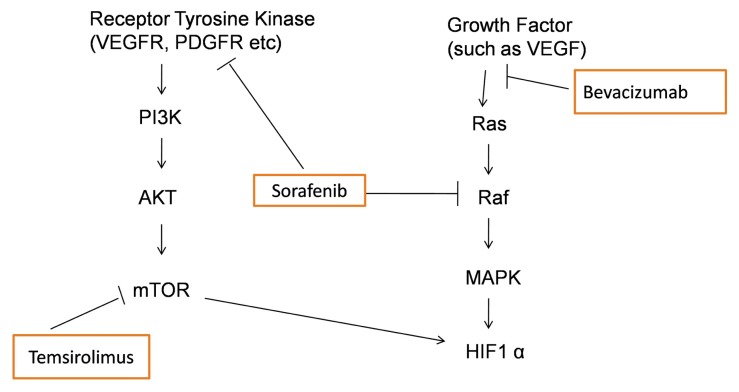
Schematic presentation of the molecular targets of different biological agents utilized in the present case.

Currently a variety of novel targeted agents have been tested in the phase II setting in ovarian cancer patients with multiple prior chemotherapy regimens [8–10]. None of these trials, however, target specific histological types. In the present case, combination therapy with sorafenib did demonstrate a partial response with a decline in the CA125 level of a heavily pretreated patient. The preclinical study also demonstrated a remarkable antitumor effect of sorafenib in OCCC [11]. Sorafenib is a multikinase inhibitor that targets the mitogen-activated protein kinase (MAPK) or Ras/Raf/ERK pathway and also inhibits other kinases (VEGFR, PDGFR) [8]. Briefly Figure 4 illustrates the molecular targets of sorafenib and other biological agents used for the treatment of the present case.

Temsirolimus, the specific small molecule inhibitor of the mammalian target of rapamycin (mTOR), inhibits the PI3K/mTOR signaling pathway by binding to the mTORC1 complex (mTOR and FK506 binding protein 12) [12,13]. Mutations in the p110α subunit of PI3K (*PIK3CA*) are often responsible for activation of the PI3K/mTOR pathway, though alterations in several other molecules including RTKs, Ras, and PTEN are also involved in the activation of this pathway [14]. Preclinical and clinical studies suggest that *PIK3CA* mutations predict the response to PI3K and mTOR inhibitors [12]. Our patient, however, did not respond to the mTOR inhibitor, temsirolimus, despite having a *PIK3CA* mutation. This is similar to our previous *in vitro* study showing that a *PIK3CA* mutation does not sensitize OCCC cells to PI3K/mTOR inhibitors [4]. This discrepancy between the studies may be due to differences in organ and subtype-specific oncogenic pathways. Though mTOR inhibitors, including temsirolimus, are being tested in different clinical trials of ovarian cancer, the inclusion criteria do not specify histology or a requirement of genetic mutation; therefore it is unlikely that these trials will yield data on predictive biomarkers for treatment selection in OCCC. Recently, we also reported that loss of ARID1A expression may affect chemosensitivity in ovarian clear cell carcinoma [15]. The present case also had an *ARID1A* mutation, which may have explained the lack of relationship between *PIK3CA* mutation and sensitivity to temsirolimus.

*PIK3CA* mutation alone may be insufficient to target therapy in OCCC. Activation of the Ras/MAPK pathway is likely important in OCCC as MAPK pathway genes are enriched in panels of OCCC signature genes [16]. Both HIF1α and HNF1B pathways activate the Ras/Raf pathway in OCCC, although other mechanisms, including activating mutations in RAF cannot be excluded [11]. The present case had high expression of HIF1α with immunohistochemistry (Figure 2C), which likely explained the activation of the Ras pathway in this case as no *Ras* mutation was identified. Moreover, phosphorylation of 4E-BP1, a downstream gene in the mTOR pathway, is regulated not only by mTORC1, but also by Ras signaling, suggesting that crosstalk occurs between the PI3K/mTOR pathway and the MAPK pathway [14]. Thus, activation of Ras signaling in OCCC may be the basis of the anti-tumor effect of mTOR inhibitors.

## 4. Conclusions

The findings of this case illustrate a potential role for targeted therapies in OCCC. Molecular profiling, including *PIK3CA* mutation, warrants further investigation in the application of targeted PI3K/mTOR inhibitors in OCCC. The Japanese Gynecologic Oncology Group (JGOG) is already conducting translational research to identify biomarkers which predict sensitivity of OCCC to the mTOR inhibitor, everolimus, (JGOG3021). Further exploration is necessary to establish the most useful clinical biomarkers for OCCC and randomized prospective trials of targeted therapies need to be conducted in the context of histology subtypes and signaling pathway mutations.

## Figures and Tables

**Table 1:** Investigated molecular profile of the current case.

ARID1A	Mutation
*PPP2R1A*	Wildtype
*PIK3CA*	Mutation
*KRAS*	Wildtype
HIF1α	High expression
